# Electroceuticals for Paralympic Athletes: A Fair Play and Classification Concern?

**DOI:** 10.1007/s40279-025-02331-1

**Published:** 2025-10-13

**Authors:** Daniel D. Hodgkiss, Shane J. T. Balthazaar, Cameron M. Gee, Ian D. Boardley, Thomas W. J. Janssen, Andrei V. Krassioukov, Tom E. Nightingale

**Affiliations:** 1 School of Sport, Exercise and Rehabilitation Sciences, College of Life and Environmental Sciences, University of Birmingham, Edgbaston, Birmingham, B15 2TT UK; 2 International Collaboration on Repair Discoveries (ICORD), Faculty of Medicine, University of British Columbia, Vancouver, BC Canada; 3 Department of Cardiology, University Hospitals Birmingham National Health Service (NHS) Foundation Trust, Birmingham, UK; 4 Department of Orthopaedics, University of British Columbia, Vancouver, BC Canada; 5 Amsterdam Rehabilitation Research Center, Reade, Amsterdam, The Netherlands; 6 Faculty of Behavioral and Movement Sciences, Vrije Universiteit Amsterdam, Amsterdam Movement Sciences, Amsterdam, The Netherlands; 7 Amsterdam Institute of Sport Science, Amsterdam, The Netherlands; 8 Division of Physical Medicine and Rehabilitation, Faculty of Medicine, University of British Columbia, Vancouver, BC Canada; 9 GF Strong Rehabilitation Centre, Vancouver Coastal Health, Vancouver, BC Canada

## Abstract

Electroceuticals such as brain computer interfaces and spinal cord stimulation (SCS) represent transformative strategies for neuromodulation. Research has demonstrated that SCS can ameliorate motor and autonomic cardiovascular dysfunctions, particularly in individuals with spinal cord injury (SCI). Notably, SCS has been shown to augment aerobic exercise performance. Owing to the nature of their injury, athletes with SCI are often predisposed to low resting blood pressure and impaired physiological responses to exercise. Therefore, some athletes intentionally induce autonomic dysreflexia (“boosting”) to gain a competitive advantage – an act banned by the International Paralympic Committee (IPC). However, the emergence of electroceuticals facilitates an alternative performance enhancement strategy that could be considered unfair without equal access opportunities for all athletes. Currently, the World Anti-Doping Agency and the IPC have not acknowledged the potential impact of electroceuticals in parasport. Herein, we present an argument that the use of SCS meets the criteria for it to be placed on the World Anti-Doping Code Prohibited List (or at the very least be monitored) because collectively: SCS can enhance sport performance, represents a potential health risk to the athlete if misused, and may violate the spirit of sport. Acute and chronic use of SCS may also lead to classification changes, and increased opportunities for athletes to intentionally misrepresent, thereby raising concerns for the IPC. The growing access to electroceuticals (e.g. via clinical trial participation or private healthcare implantation) more than ever increases the likelihood of an athlete using SCS to gain an unfair advantage in parasport.

**Supplementary Information:**

The online version contains supplementary material available at 10.1007/s40279-025-02331-1.

## Key Points

**Table:** 

Electroceuticals, namely spinal cord stimulation (SCS), have the potential to enhance performance in parasport and influence classification, thereby falling within various International Paralympic Committee (IPC) policies (e.g. The IPC Anti-Doping Code, The Classification Code, The Medical Code, and The Policy on Sport Equipment).
Evidence indicates that SCS meets the requirements for the World Anti-Doping Agency (WADA) to consider its place on the Prohibited List, as it meets at least two—and possibly three—of the following criteria: (1) the potential to enhance or enhances sport performance; (2) represents a potential health risk to the athlete if misused, and (3) may violate the spirit of sport.
We propose several considerations for the IPC and WADA to get ahead of the impending use of electroceuticals in para-athletes: (1) require athletes to declare whether they have an electroceutical and will use it during competition; (2) develop guidelines for monitoring electroceutical use immediately prior to and during competition; (3) monitor long-term use to assess whether athletes have undergone a period of neuroplasticity whereby improvements in function (i.e. by using SCS to augment training adaptations) may warrant more frequent re-classifications, and (4) start to educate para-athletes and athlete support personnel now so that they are aware of the ethical and practical implications of electroceuticals ahead of future regulation implementation.

## Introduction

Electroceuticals are broadly defined as devices that therapeutically modulate the nervous system to ameliorate impaired functions (i.e. neuromodulation). Several strategies to enhance functions in individuals with neurological disorders [e.g. spinal cord injury (SCI), cerebral palsy, multiple sclerosis, multiple systems atrophy, Parkinson's disease] are emerging. One such example is a brain–computer interface (BCI), which involves the formation of a ‘digital bridge’ between cortical and epidural implants in a manner designed to restore movement [1, 2]. BCIs have received extensive media interest across the last few years, mainly focused on Elon Musk’s Neuralink device being successfully implanted into two human participants as part of a clinical trial [3]. Restoring lost functions via such methods may not only contribute to improving health-related quality of life but could also have 'knock-on' consequences for improving physiological responses during exercise. This Current Opinion explores how electroceuticals, with a specific focus on spinal cord stimulation (SCS), could enable individuals with neurological disorders to enhance performance during training and competition. We introduce the topic of ‘neuro-doping’, which has only received interest in the wider, able-bodied population thus far. The controversial use of technological aids to enhance performance has been questioned before (e.g. the use of highly specialised prosthetic limbs that provide a performance advantage over other athletes), resulting in political ramifications [4]. We propose potential considerations on how organisations such as the International Paralympic Committee (IPC) and the World Anti-Doping Agency (WADA) may manage the use of electroceuticals in the future to monitor potential misuse, prevent unfair advantages and mitigate potential harm.

## What is Spinal Cord Stimulation?

Research on SCS to treat secondary complications associated with SCI has flourished in recent years [5]. SCS is predominantly formed of two strategies: epidural and transcutaneous. Epidural SCS consists of an implantable electrode array connected to an indwelling pulse generator that provides tonic electrical stimulation to the spinal cord. This is typically controlled by a user-friendly, hand-held programming device or mobile app. In contrast, transcutaneous SCS is administered via skin surface electrodes that are connected to a constant current stimulator. Whilst traditional, research-grade transcutaneous SCS devices have required a trained operator, recent devices are being commercialised with relatively easy-to-use interfaces—by the end-user—in comparison with past technology.

SCS has demonstrated a remarkable augmentation of motor–sensory and autonomic cardiovascular functions at rest [5–8]. Specifically, improvements in hand/arm functions (e.g. reaching), trunk stability, seated posture, and trunk and back muscle activity with SCS have been noted in individuals with SCI [9, 10]. Motor benefits with SCS have also been demonstrated in other neurological conditions [11–14]. With regards to autonomic cardiovascular function, research has shown that activation of the sympathetic circuitry in the lower thoracic segments (i.e. T10–T12), known as the ‘haemodynamic hotspot’, results in a well-controlled, closed-loop baroreflex-like increase in blood pressure (BP) [15]. More recently, through a series of clinical studies, Phillips et al. [16] elegantly demonstrated that a closed-loop epidural SCS system can trigger robust pressor responses to mitigate and treat both resting and orthostatic hypotension in individuals with higher neurological levels of SCI. Over time, daily use of the implant to reduce complications associated with hypotension led to improvements in health-related quality of life and the alleviation of other secondary complications (e.g. spasticity, disrupted sleep, bowel dysfunction) [16]. Given the evidence demonstrating that SCS can effectively modulate BP at rest, it is therefore unsurprising that such changes map onto exercise-related autonomic functions [8].

## Neuro-Doping, Fair Play and Athlete Health

Doping is a significant issue in sport. There is ongoing concern that some athletes cross the line and use prohibited substances and/or methods to gain an unfair performance advantage during competition. To maintain fair play and protect athlete health, individuals competing in sport are required to adhere to the World Anti-Doping Code [17]. Other authorities (e.g. the IPC) typically adopt the information presented in the World Anti-Doping Code within their own regulations (e.g. The IPC Anti-Doping Code [18]). Therefore, whilst discussions hereafter may focus on the World Anti-Doping Code, we highlight that these points also relate to other authorities (e.g. the IPC).

The World Anti-Doping Code outlines that for a substance or method to be placed on the Prohibited List, it needs to meet *two* of the following *three* criteria: (1) it has the potential to enhance or actually enhances sport performance; (2) it represents an actual or potential health risk to the athlete, and (3) it violates the spirit of sport. Once a substance or method is deemed prohibited, a decision must then be made on whether it should be prohibited in both competition and training, only in competition, or only in certain sports. Alternatively, WADA may choose to place such novel methods within a monitoring program until further evidence corroborates its effects.

A relatively new concept in the doping field is the act of ‘neuro-doping’, which involves the application of neuromodulation to gain a performance advantage in sport. This term is akin to ‘techno-doping’, which is an emerging concern in both para- and non-parasport, referring to the use of technology to enhance performance [19, 20]. Note, whilst both ‘neuro-doping’ and ‘techno-doping’ contain the term ‘doping’, we acknowledge that these methods (when not in the context of gaining a competitive advantage in an event) do not automatically defer to the doping field. These may also be considered as acts that require consideration by authorities other than WADA (e.g. the IPC during the classification process).

The majority of discussions on whether neuro-doping methods should be on the Prohibited List so far have been limited to their use in able-bodied athletes [21–25], owing to their ability to alter psychophysiological responses during exercise and enhance performance. Research in able-bodied athletes has predominantly indicated that transcranial direct current stimulation (tDCS) can modulate motor function, cognitive performance, or perceptions of fatigue [26–30]. Despite this, tDCS and other forms of neuromodulation are not currently prohibited by WADA.

In the following sections we discuss how SCS meets the three aforementioned WADA criteria used to determine what constitutes doping, along with a discussion on how SCS may impact the classification process governed by the IPC. We also present an alternative viewpoint on whether SCS should be prohibited in para-athletes, given that the technology is designed to augment motor and autonomic dysfunctions, rather than enhance performance beyond normal (e.g. as is the case for individuals without such dysfunctions). Yet, the ability to improve performance relative to other para-athletes with similar classifications represents an unfair advantage, particularly as these therapies may not be accessible to all (e.g. athletes from under-developed countries or with financial hardship).

### Spinal Cord Stimulation has the Potential to Enhance or Actually Enhances Sport Performance

In many individuals with SCI, especially those with higher neurological levels of injury (i.e. at or above T6), exercise performance is limited not only by motor impairments such as trunk instability and/or upper-limb muscle control and spasticity but also by cardiovascular dysfunction. The latter typically manifests as a lower resting BP as well as a lower peak heart rate, reduced left-ventricular stroke volume, and thus, maximal cardiac output, which ultimately limits aerobic exercise performance [31–34]. It should be emphasised that many athletes with SCI are predisposed to BP instability and often present with resting hypotension [35], which compromises their ability to generate a sufficient pressor response during aerobic exercise.

In addition to motor and cardiovascular limitations, thermoregulation further compounds these challenges [36, 37]. Individuals with SCI have impaired blood flow redistribution to the skin [38, 39], smaller or absent sweat rates [40, 41], a disturbed temperature perception and comfort [42], and higher core temperatures during exercise [36–41, 43], in comparison with non-injured individuals. Such impairments to essential heat loss mechanisms predispose individuals with SCI to an earlier onset of exertional heat stress and therefore impaired performance during prolonged events in both hot and temperate environments [44].

Such cardiovascular impairments, due to abnormal autonomic nervous system (ANS) control, are also reported in other neurological conditions (e.g. multiple sclerosis and muscular dystrophy), which restrict exercise performance by limiting perfusion pressure and result in insufficient blood flow to working muscles [45, 46]. To overcome these ANS-mediated cardiovascular impairments that limit aerobic exercise capacity, some athletes with higher neurological levels of SCI may intentionally induce autonomic dysreflexia (AD), known as ‘boosting’. This is a practice whereby (non-)noxious stimuli (e.g. via intentional bladder or bowel distention, pin pricks, excessive lower-body compression, twisting and/or sitting on the scrotum, or self-inflicted injury [47]) below the level of injury elicit a sympathetic response to increase BP. In turn, this alters physiological responses to exercise and enhances performance [48–52]. Boosting was originally banned by the IPC in 1994 primarily owing to the life-threatening health consequences associated with eliciting AD (e.g. increased risk of cardiac events, aneurysms, cerebral haemorrhage, seizures and death) [53] but also owing to research demonstrating its performance-enhancing effects [49]. Therefore, some para-athletes may consider alternative performance enhancement strategies to elicit similar physiological responses but without the need to self-harm.

SCS has been shown to be a promising strategy for addressing these physiological limitations to enhance performance. A case study on an individual with chronic tetraplegia reported that epidural SCS increased BP and oxygen pulse (a surrogate for stroke volume), augmenting peak oxygen uptake (V̇O_2peak_) by up to 26% during a graded arm-crank ergometry test [54]. Similarly, a case series found that epidural and transcutaneous SCS modulated cardiovascular responses at rest and enhanced submaximal arm-crank exercise performance [55]. Both forms of SCS significantly extended time to exhaustion, with SCS enabling participants to exercise for up to 19 min longer at the same power output than a session with sham SCS. This equated to an average 48% (range 32–80%) improvement in performance. Ratings of perceived exertion tended to be lower throughout all exercise trials with SCS, relative to sham SCS. Notably, one participant receiving transcutaneous SCS had a $\dot{V}$O_2peak_ on par with elite athletes with SCI [56], thus highlighting the potential for athletes to modulate their physiological responses and enhance their performances through the use of electroceuticals.

SCS also offers further potential benefits. Self-reported improvements in temperature regulation with SCS have been noted [57–61], which could have implications for managing thermal stress during performances. SCS applied over the cervical spinal cord has demonstrated improvements in upper-limb motor function [10], which could be useful for para-athletes requiring fine motor skills (e.g. wheelchair basketball or rugby). SCS can also reduce spasticity [62–64]. A study on two paraplegic swimmers demonstrated that transcutaneous SCS, alongside lower-limb functional electrical stimulation (FES), eliminated spasticity in the lower extremities for up to 4 hours beyond the duration of the session [65]. Both participants reported that stimulation-assisted swimming was comfortable, enjoyable and they would like to use such a device for recreational training and rehabilitation in the future. Notably, the combination of transcutaneous SCS with FES over a prolonged period of training enhanced lap time performance in comparison with swimming with FES alone.

Whilst literature on the performance-enhancing effects of SCS is still emerging, it is important to note that regulation can occur in the absence of high levels of evidence [i.e. randomised controlled trials (RCTs)]. Such examples are presented in Table 1. Ultimately, these precedents emphasise that sporting bodies, such as the IPC and WADA, have historically relied on physiological plausibility, health risks, data from individual cases and concerns over fairness, rather than waiting for conclusive RCT data when regulating emerging substances and technologies. Therefore, a proactive stance to the emergence of electroceuticals and the threat they pose to parasport is a timely concern.

**Table 1:** Previous examples of substance or method prohibition by the IPC or WADA based on preliminary evidence or plausibility of their performance-enhancing effects

(1) Recombinant human erythropoietin was banned in 1990 by the IPC and the International Olympic Committee (IOC) shortly after its commercial availability. Despite no RCT-level evidence in athletes, it was banned on the basis of the physiological plausibility of enhancing performance via erythropoiesis [66].
(2) Blood doping was prohibited by the IOC in 1985 on the basis of small, non-randomised trials demonstrating that blood transfusions could increase maximal oxygen consumption [67, 68]. Regulation occurred on the basis of an anticipated advantage and ethical concerns.
(3) Gene doping was banned by WADA in 2003, despite no evidence of its use in sport nor RCTs demonstrating its performance-enhancing potential. Its ban was also anticipatory, considering the perceived threat to fairness and athlete safety.
(4) A further case is the controversy surrounding Oscar Pistorius’s use of carbon fibre prosthetic blades. In 2008, the International Amateur Athletics Federation initially ruled him ineligible to compete against able-bodied athletes on the grounds that his prostheses could provide a mechanical advantage. This decision was made in the context of limited and contested scientific evidence, yet the perceived performance enhancement and violation of the spirit of sport was sufficient for regulation at that stage.

### Spinal Cord Stimulation Represents a Potential Health Risk to the Athlete

Similar to boosting, there is scope to consider how electroceuticals could be dangerous. Despite over 70 years of data supporting the safety of epidural SCS for patients with chronic pain, individuals with neurological conditions present with unique comorbid conditions (e.g. skeletal muscle atrophy, osteoporosis, higher risk of infections, falls and fractures) that may contribute to a different spectrum of possible adverse events (AEs). Moreover, the daily dose of stimulation delivered with epidural SCS to elicit functional changes in individuals with SCI is highly variable, as a result of the preferences of the individual and the heterogeneity of their injury, and is far greater than that delivered in patients with pain [69]. A recent scoping review reported that approximately a quarter of unique studies using either invasive or non-invasive SCS reported one or more AEs in individuals with SCI, and generally risk and safety was poorly reported across the literature [5]. Importantly, there is a big question over whether athletes using SCS could self-harm (akin to boosting [48]) by using the technology above and beyond what is recommended, and without supervision. Evidence has also shown that SCS may result in episodes of AD [70, 71]. Misuse of electroceuticals outside of their tested capacities in clinical research could result in unforeseen safety issues. Despite this, preliminary evidence indicates that both SCS modalities are safe when used in closely monitored clinical trials in individuals with SCI [10, 16, 69]. When administered in research studies, SCS can treat or prevent AD events [72, 73] or hypotension [15, 16, 54, 55, 74–78], thereby minimising the impact of these detrimental secondary conditions rather than exacerbating them. An important distinction is that in research studies, SCS results in a safe normalisation of BP, in contrast to ‘boosting’, which results in an uncontrollable and often unpredictable rise in BP [49].

There are also several other unknowns regarding safety considerations in para-athletes for implantable electroceuticals. For example, collisions during events such as wheelchair rugby or basketball could cause lead migration or hardware failure that requires the need for subsequent invasive corrective surgery. Another emerging concern is the potential for cyber-security vulnerabilities, with the possibility for electroceuticals to be hacked or exploited [79, 80]. These considerations clearly meet the broad spectrum of athlete health criteria outlined in the IPC Medical Code [81], emphasising the need for guidelines and research to ensure the safe management of SCS in athletic contexts, particularly at the elite (i.e. Paralympic) level.

### Spinal Cord Stimulation May Violate the Spirit of Sport

Perhaps one of the most fundamental violations of the spirit of sport is that individuals who use an electroceutical may gain an unfair competitive advantage over those who do not [23]. This concept threatens the key spirit of sport values such as ‘respect for self and other participants’, ‘community and solidarity’, ‘ethics, fair play and honesty’ and ‘character and education’ [17]. Consider a situation where an athlete narrowly misses out on placing with a medal to someone who had used an electroceutical to gain an advantage. This would clearly damage the integrity of fair play and challenge what the spirit of sport criterion is designed to protect against. This concern would be further amplified if access to electroceuticals was determined by financial means, which is potentially disadvantageous to athletes from low-income backgrounds and thereby raises an equality issue.

### The IPC Classification Concern

One way for SCS to be used unfairly in parasport is during the classification process. To be eligible to compete in the Paralympic Games and World Championships, individuals must undergo classification to determine the type and degree of impairment [82]. As stated in the IPC Classification Code [83], the purpose of this process is to promote fair and meaningful competition by minimising the impact of athletes’ impairments on the outcome of competition so that the outcome is determined by factors other than impairment. Classification is predominantly focused on motor deficits, for which there is scope for athletes to alter with SCS. As such, electroceutical use is susceptible to intentional misrepresentation [84]. Also known as ‘classification cheating’, intentional misrepresentation constitutes a deliberate attempt to misrepresent an athlete’s skills, abilities or impairment to gain a competitive advantage through the classification process [85]. The emergence of electroceuticals potentially represents an additional means of intentional misrepresentation, as individuals who use electroceuticals may choose not to declare—or use—their device during classification despite the performance advantage it may provide when operational during competition.

## Wider Considerations for Use of Electroceuticals in Parasport

Private healthcare companies such as Verita Neuro, which has facilities in Calgary (Canada), Guadalajara (Mexico) and Bangkok (Thailand), are currently performing implantation procedures so that the epidural stimulation technology is available to those who can afford it (upwards of US $100,000). Currently, their website advertises that at least 200 individuals have been successfully implanted with an epidural stimulator. Portable transcutaneous SCS devices possibly represent a more realistic option for use in the community owing to their relatively less invasive nature; however, there is still a sizeable financial cost for these devices (approximately US $15,000–40,000). To this end, companies such as Onward (ARC^EX^), SpineX (SCONE) and Aneuvo (ExaStim) are seeking to commercialise their portable transcutaneous SCS devices by demonstrating their safety and efficacy in clinical trials (e.g. NCT05111093, NCT05941819, NCT05044923, NCT04994886, NCT04697472, ISRCTN17856698, NCT06313515). Moreover, a commercially available neuromuscular electrical stimulation device has recently been shown to improve autonomic function in an individual with SCI, presenting an opportunity for devices already on the market to be used [86]. These costs and access opportunities, however, arguably place athletes from low-to-middle income countries at an unfair disadvantage in comparison with those from Westernized countries, which could be considered a violation of the spirit of sport.

Outside of private implantations, a surge in epidural SCS clinical trial registrations over the last few years indicates that there are greater opportunities for individuals to enrol in studies and undergo implantation. As of January 2025, we identified 26 clinical trials (*N* = 508 participants) featured on multiple online registries that have explored (or are exploring) the effects of epidural SCS on hand or trunk function and/or BP regulation specifically in individuals with SCI (Supplementary File 1). Many more trials are investigating the effects of epidural SCS on other outcomes (e.g. locomotion, lower extremity motor function, spasticity, and bowel, bladder and sexual function), or in individuals with other neurological conditions. Once a trial is completed, it is assumed that participants would be given the opportunity for their device to be reconfigured to target other indications. A similar case has occurred in the Paralympic field previously. Jennifer French, who sustained a C6/7 SCI in 1998, became the first US woman to receive an implanted stand and transfer neuromuscular electrical stimulation system as part of a clinical trial in 1999. This system was later upgraded in a subsequent trial in 2010. Her participation involved major surgery, intensive rehabilitation and close collaboration with clinicians to optimise stimulation patterns and electrode configurations. As a result of participating in the clinical trial, French noted remarkable improvements in standing and stepping, cardiovascular function (i.e. blood flow re-distribution), trunk stability and function in activities of daily living. The device was likely a crucial component contributing to her athletic development, which culminated in a silver medal for Team USA in para-sailing at the 2012 Paralympic Games [87].

Ultimately, the odds of an athlete choosing to enrol in a trial are higher than ever. The odds of someone becoming an athlete who already has a device is also a possibility. It is important to note that we do not envisage an athlete using SCS (or undergoing implantation) solely for the purpose of enhancing their performance. Any ergogenic benefit is likely to be secondary to a device being used to alleviate the complications associated with the neurological dysfunction that detrimentally impacts their health-related quality of life.

A recent survey of 223 individuals with SCI in North America revealed significant interest in the use of SCS, with 61% and 80% expressing interest in using epidural and transcutaneous SCS, respectively, and 91% indicating readiness to follow a specific training protocol [88]. Whilst this survey focused on non-athletes, anecdotal insights from conversations among the parasport community suggest athletes are already aware of the potential of electroceuticals. Many para-athletes express openness to using such devices to provide them with a competitive advantage in their respective sports. Perspectives from such individuals are presented in Supplementary File 2, offering insight into the evolving attitudes towards the integration of neuromodulation in competitive settings.

An alternative viewpoint for consideration regarding electroceutical use in parasport is to allow free use by athletes, as it may be argued that SCS is attempting to ‘normalise’ physiological responses by alleviating neurological dysfunctions that detrimentally impact quality of life. This would be different to able-bodied athletes using substances or methods to enhance performance beyond normal. Therefore, this raises an ethical quandary as it would not be appropriate to say that an athlete cannot compete because they use SCS during their daily life. Yet, athletes who decide to use an electroceutical during competition may experience acute performance benefits that unfairly outmatch the physiological adaptations other para-athletes of similar classification have achieved through training [23]. If not monitored or managed by governing bodies, this quandary may also result in athletes who do not want to use an electroceutical, feeling pressured to do so to be competitive. Moreover, another argument against the open use of electroceuticals is that individuals may respond in different ways, with some athletes receiving more of a benefit than others. Indeed, the recent UP-LIFT study demonstrated a high degree of heterogeneity in hand function improvement following an intervention with transcutaneous SCS [10].

## Suggestions for the Monitoring and Management of Electroceutical Use in Parasport

Currently, there is no specific guidance to regulate the use of electroceuticals in parasport, but their potential use (or misuse) by athletes warrants attention. Some para-athletes, including those who have sought to enhance their performance without due regard for fair play previously (e.g. those who have boosted or intentionally misrepresented), may be more likely to take advantage of these opportunities and use electroceuticals beyond what is recommended. Without clear guidelines and safeguards for athletes to adhere to, it could be argued that electroceuticals represent a new form of technological doping to enhance exercise performance, thus jeopardizing fair play and the integrity of parasport competition.

### IPC Policy on Sport Equipment

To address this, the IPC could consider how electroceuticals align with their Policy on Sport Equipment [89]. Section 3.1.4 on Physical Prowess states that ‘Human performance is the critical endeavour to the sport performance, not the impact of technology and equipment’. This is expanded in Sect. 3.2 of the policy, in which it states, ‘Equipment that results in sport performance not primarily being generated by the athlete’s own physical prowess but being generated by automated, computer aided, or robotic devices is prohibited in IPC Sanctioned Competitions and Events, and at Paralympic Games’. Therefore, by this definition, electroceuticals could be prohibited by the IPC if they are shown to enhance performance.

### The Difficulty in Proving Intent

One significant challenge is the difficulty of proving a para-athlete’s intention to gain an unfair advantage with SCS. This dilemma is what contributed to the removal of boosting from the IPC Anti-Doping Code, primarily owing to the burden of proving intent to induce AD. A subsequent Position Statement on Autonomic Dysreflexia and Boosting was published to avoid this burden [90]. However, the difficulty in proving intent to boost via AD is significantly different to how difficult it may be to prove intent with SCS. Specifically, enhancing performance with SCS is inherently more intentional, as an athlete or coach will physically need to turn on the device. In contrast, there are clear mechanisms in which athletes may unintentionally boost (e.g. by forgetting to empty their bladder which results in overdistension or having a slight change in positioning that pinches the skin etc.) [50, 91].

The difficulty of proving intent is also relevant when one considers that SCS may be required to manage secondary complications. This situation appears similar to Therapeutic Use Exemptions provided by WADA, in which athletes are permitted to use substances or methods to ensure they can compete in a proper state of health but without gaining an unfair competitive advantage. Athletes who have an electroceutical could be asked to provide medical documentation to justify their need for an electroceutical, and their use within competition could theoretically be regulated. Alternatively, IPC or WADA personnel could simply request that all athletes declare whether they have an electroceutical and be asked to keep self-report logs of when it has been used (or usage logs within the device itself could be used). Athletes who are caught using an electroceutical without permission (or who are not on an approved list) could be sanctioned appropriately. An analogous situation in doping is that of strict liability. Here, athletes can be sanctioned for the mere presence of a substance in their system, without the sanctioning body having to prove any intent to use or gain a performance advantage on the part of the athlete. Alternatively, the IPC could consider how electroceutical use may breach the Code of Conduct for Athletes laid down in the IPC Code of Ethics [92]. Here, it states ‘It is recognised that athletes may have significant medical conditions that require treatment, but the use of any technique or medication whose sole purpose is sport performance enhancement while being detrimental or potentially detrimental to health will not be tolerated’.

### Policing and/or Monitoring of Electroceuticals

Electroceuticals such as epidural SCS, however, could cause potential policing issues given their inherent nature of being implanted and therefore more easily concealable. Stimulation can be administered via a hand-held programmer or mobile phone app, which an athlete could operate independently or with the assistance of a coach or support staff. In practice, the stimulator could be turned on before an event and maintained throughout whilst the controller/programmer is handed off to a coach or left off site, thereby making real-time detection or policing extremely difficult. Conversely, transcutaneous SCS, which is administered using skin-surface electrodes and visible leads, would be harder to conceal during competition. In practice, the device would need to be with the individual. However, policing transcutaneous SCS may become more difficult over time with technological advancements facilitating the integration of stimulation electrodes into garments [93]. WADA also runs a monitoring program which includes substances that are not on the Prohibited List but that they wish to monitor to detect patterns of misuse in sport. Given the observed performance-enhancing effects of SCS [54, 55], WADA could at the very least monitor for potential misuse in parasport.

The monitoring of electroceutical use to augment cardiovascular responses and enhance performance is also complicated by the fact that currently the classification system does not consider the degree of para-athlete autonomic impairment. This is despite previous calls and evidence suggesting that cardiovascular dysfunction should be considered [94, 95]. Ignoring this within the classification process could enable athletes with autonomic dysfunction to enhance their physiological responses during performances and unfairly compete against others with more profound autonomic dysfunction and without an electroceutical. One solution could be for para-athletes to be classified with or without an electroceutical (whichever they intend to use during competition). However, the current classification system would only capture the motor benefits and not the notable cardiovascular benefits that also have a profound performance advantage. Importantly, if an athlete knowingly chooses not to use their device when undergoing classification, but does use it during subsequent competition, this raises substantial concerns as this represents a form of intentional misrepresentation. Given the threat to fair play, at the very least the IPC could consider mandating acknowledgement of electroceutical use within the classification process itself.

Importantly, we know that SCS has the potential to enhance performance acutely and could therefore be used solely in competition. An ‘electroceutical passport’, akin to the biological BP passport previously recommended to monitor boosting [95], may be an effective starting point to regulate athlete use of electroceuticals across competitions. Recent work has reported that repeated use of SCS has the potential to alter motor-sensory scores assessed during an International Standards for Neurological Classification of Spinal Cord Injury (ISNCSCI) exam [96–98]. Whilst there is currently no longitudinalevidence on performance improvements with repeated use of SCS during training, ongoing clinical trials (ISRCTN17856698, NCT06313515) are attempting to explore this. As an example, it is hypothesised that chronic use of SCS during daily training would induce a greater volume load of the cardiac chambers than would occur without SCS, which, over the longer term, would result in functional and structural cardiac adaptations, ultimately improving performance. More frequent classifications could take place to track any long-term functional improvements resulting from electroceutical use.

### Education on Electroceuticals

Education is another key component. Experts have identified threats to integrity within the classification process as a high priority for education delivered to both para-athletes and athlete support personnel [99]. Clean sport programs should address the potential use of electroceuticals to threaten fair play. Whilst there is a risk that this would further raise awareness and para-athlete interest in electroceuticals, the potential health risks to athletes are of utmost concern and call for preventative education programs to begin now alongside the timely implementation of guidelines and safeguards by governing bodies.

### Recommendations

Whilst continued work (i.e. RCTs) is required to corroborate current findings on the effectiveness of electroceuticals for enhancing exercise performance, we propose several considerations for the IPC and WADA (Table 2) to get ahead of their impending use by para-athletes.

**Table 2:** Considerations for the International Paralympic Committee and the World Anti-Doping Agency on electroceutical use in parasport

(1) Require athletes to declare whether they have an electroceutical and will use it during competition.
(2) Develop guidelines for monitoring electroceutical use immediately prior to and during competition.
(3) Monitor long-term use to assess whether athletes have undergone a period of neuroplasticity whereby improvements in function (i.e. by using their device to augment training adaptations) may warrant more frequent re-classification.
(4) Educate para-athletes and athlete support personnel now so that they are aware of the ethical and practical implications of electroceuticals ahead of future regulation implementation.

## Conclusions

Whilst there remain many unknowns with regards to the safety of electroceuticals in parasport, existing evidence suggests that they have the potential to enhance sport performance, could put athletes at risk of harm if misused, and may also violate the spirit of sport. As such, their use meets at least two—if not all three—of the requirements for WADA to prohibit their use. This concern also extends beyond anti-doping regulations alone. Electroceuticals have direct implications for multiple IPC policy areas, including the IPC Anti-Doping Code, Classification Code, Medical Code and their Policy on Sport and Equipment. Therefore, we call on researchers, the IPC and WADA to consider the potential ethical and health consequences of athletes using electroceuticals to enhance their performances, even in cases where performance enhancement is a secondary effect to alleviating the many complications and symptomatology associated with neurological disorders. Gaining an unfair and unacknowledged performance advantage with the use of an electroceutical is of significant concern, as is the misuse of such technologies that may put athletes at risk of significant health consequences. The protection of fair and safe play in parasport requires early, urgent collaborative action to establish guidelines, monitor use, and educate individuals on electroceutical use.

## Supplementary Information

Below is the link to the electronic supplementary material.

Supplementary file1 (DOCX 25 KB)

Supplementary file2 (DOCX 15 KB)
