# Supplementary material for: Electroceuticals for Paralympic Athletes: A Fair Play and Classification Concern?
Source: Sports Med. 2025 Oct 13;56(2):315–25. doi: 10.1007/s40279-025-02331-1 (PMC12982197; doi:10.1007/s40279-025-02331-1)
Supplement: Supplementary file 1 — Supplementary file1 (DOCX 25 KB) [file 40279_2025_2331_MOESM1_ESM.docx]

Using the terms “Spinal Cord Injury” and “Spinal Cord Stimulation”, the following clinical trial databases were searched:

- ClinicalTrials.gov
- International Standard Randomised Controlled Trial Number (ISRCTN)
- Australian and New Zealand Clinical Trials Registry (ANZCTR)
- European Clinical Trials Register (EU-CTR)
- Brazilian Clinical Trials Registry
- Hong Kong University Clinical Trial Register
- International Clinical Trials Registry Platform (ICTRP) was also searched to identify trials conducted in countries that may not appear under the above databases.

Identified studies were examined against the following criteria:

1. Participants were implanted as part of the study. This was included to prevent duplication of participants in our calculation.
2. Status of the study was either completed, recruiting, not recruiting, not yet recruiting, or unknown. Any study that was identified as withdrawn or terminated was not included.
3. Outcomes must include an assessment of hand or trunk motor function, or overall balance test, or an assessment of blood pressure regulation.

**Table 1.** Clinical trial identifiers for studies investigating the effects of implanted epidural spinal cord stimulation on trunk motor function and/or overall balance, or autonomic cardiovascular function outcomes, in individuals with spinal cord injury.

| **Trial Number** | **Study Title** | **Study Status** | **Target Recruitment** | **Location** | **Relevant Outcomes** |
| --- | --- | --- | --- | --- | --- |
| NCT02037620 | Recovery of Cardiovascular Function With Epidural Stimulation After Human Spinal Cord Injury | C | 5 | University of Louisville | Mean arterial blood pressure |
| NCT02313194 | Spinal Cord Neuromodulation for Spinal Cord Injury | NR | 12 | University of California | Arm/hand function via formal motor testing |
| NCT02329652 | Multi-functional Neuroprosthetic System for Restoration of Motor Function in Spinal Cord Injury | R | 30 | MetroHealth Medical Center, Cleveland | Activities of Daily Living Abilities Test in response to receiving implanted networked neuroprosthetic system for hand, arm, and trunk function. |
| NCT02592668 | Spinal Cord Injury Epidural Stimulation | C | 2 | Mayo Clinic | Change in sitting balance via functional reach test  Duration of time for which subject can sit unassisted on the edge of a mat table  Change in body thermoregulatory capacity as measured by thermoregulatory sweat testing |
| NCT02936453 | STIMO: Epidural Electrical Simulation (EES) With Robot-assisted Rehabilitation in Patients With Spinal Cord Injury | NR | 10 | Ecole Polytechnique Fédérale de Lausanne | Static balance via Berg Balance Scale |
| NCT03026816 | Epidural Stimulation After Neurologic Damage | R | 100 | University of Minnesota | Systolic blood pressure measured during epidural stimulation (continuous) |
| NCT03364660 | Task-specific Epidural Stimulation Study | R | 36 | University of Louisville | Recovery of autonomic control of cardiovascular function as assessed by cardiovascular assessments  Recovery of voluntary movement as assessed by functional movement assessments |
| NCT03452007 | SPARC Bladder Mapping and Training Study | R | 10 | University of Louisville | Changes in systolic blood pressure |
| NCT03924388 | Spinal Cord Stimulation and Autonomic Response in People With SCI | U | 46 | University of Calgary | Changes in episodic blood pressure acutely (beat-to-beat) and over a 24-hour period (ambulatory blood pressure monitoring) |
| NCT04105296 | Epidural Stimulation After Spinal Cord Injury | U | 5 | McGuire VA Medical Center | Changes in systolic and diastolic blood pressure |
| NCT04193709 | Recovery of Bladder and Sexual Function After Human Spinal Cord Injury | R | 70 | University of Louisville | Change from baseline in systolic blood pressure over a 24-hour period |
| NCT04632290 | Brain-controlled Spinal Cord Stimulation in Patients With Spinal Cord Injury | U | 3 | Ecole Polytechnique Fédérale de Lausanne | Static balance via Berg Balance Scale  Upper limb neurobiomechanics (average range of movement, amplitude of electromyography activity during upper limb movements) |
| NCT04782947 | Epidural Stimulation and Resistance Training After SCI | R | 20 | Hunter Holmes McGuire VA Medical Center, Richmond | Changes in systolic and diastolic blood pressure |
| NCT04879862 | Locomotor and Bladder Function in Individuals With Acute Spinal Cord Injury | R | 16 | University of Louisville | Laboratory and ambulatory blood pressure monitoring |
| NCT04894734 | Spinal Cord Stimulation (SCS) for Spinal Cord Injury (SCI) | R | 30 | Duke University | Motor recovery (upper extremity motor score) |
| NCT04994886 | Restoring Hemodynamic Stability Using Targeted Epidural Spinal Stimulation Following Spinal Cord Injury | R | 4 | Lausanne University Hospital | Trunk stability during systematic reaching movements  Beat-to-beat blood pressure monitoring during an orthostatic head-up tilt test  Autonomic Dysfunction Following Spinal Cord Injury (ADFSCI) questionnaire |
| NCT05044923 | Restoring Hemodynamic Stability Using Targeted Epidural Spinal Stimulation Following Spinal Cord Injury | U | 4 | Lausanne University Hospital | Trunk stability during systematic reaching movements  Beat-to-beat blood pressure monitoring during an orthostatic head-up tilt test  Autonomic Dysfunction Following Spinal Cord Injury (ADFSCI) questionnaire |
| NCT05111093 | Epidural Electrical Stimulation to Restore Hemodynamic Stability and Trunk Control in People with Spinal Cord Injury | R | 20 | Ecole Polytechnique Fédérale de Lausanne | Beat-to-beat blood pressure monitoring during an orthostatic head-up tilt test |
| NCT05433064 | Spinal Cord Stimulation for Spinal Cord Injury Patients - Regain Walk and Alleviate Pain | R | 12 | Buddhist Tzu Chi General Hospital | Trunk stability via force plate and Berg Balance Scale) |
| NCT05644171 | RESTORES Trial: RESToration Of Rehabilitative Function With Epidural Spinal Stimulation | R | 3 | Tan Tock Seng Hospital | Volitional motor function assessments  Haemodynamic control assessments |
| NCT05665998 | Brain Controlled Spinal Cord Stimulation in Participants With Cervical Spinal Cord Injury for Upper Limb Rehabilitation | R | 3 | Ecole Polytechnique Fédérale de Lausanne | Arm and hand function [Graded and Redefined Assessment of Strength Sensibility, and Prehension (GRASSP) score; Action Research Arm Test (ARAT); Capabilities of the Upper Extremity Test (CUE-T); Range of Motion; Grasp force; Pinch force] |
| NCT05690074 | Epidural Stimulation in Chronic Spinal Cord Injury Patients | R | 5 | University Hospital, Motol | Trunk control stability test  Change in blood pressure during a head-up tilt test |
| NCT06243952 | Brain Controlled Spinal Cord Stimulation in Participants with Spinal Cord Injury for Lower Limb Rehabilitation (Think2Go) | R | 3 | Ecole Polytechnique Fédérale de Lausanne | Static balance via Berg Balance Scale |
| NCT06410001 | CE-STAND: Cervical Epidural STimulation After Neurologic Damage | NYR | 36 | University of Minnesota | Change in systolic and diastolic blood pressure and heart rate during tilt table testing  Trunk stability via the Multidirectional Reach Test |
| NCT06455137 | Epidural Electrical Stimulation for Spinal Cord Injury Patients and Corticospinal Motor Circuit Improvement | R | 20 | Buddhist Tzu Chi General Hospital | Static balance via Berg Balance Scale |
| NCT06533969 | Assessment of Neural Signals for the Control of Assistive Devices | R | 3 | University of Miami | Hand motor function (via Toronto Rehabilitation Institute Hand Function Test) |

C, completed; NR, not recruiting; NYR, not yet recruiting; R, recruiting; U, unknown.
