# Supplementary material for: Electroceuticals for Paralympic Athletes: A Fair Play and Classification Concern?
Source: Sports Med. 2025 Oct 13;56(2):315–25. doi: 10.1007/s40279-025-02331-1 (PMC12982197; doi:10.1007/s40279-025-02331-1)
Supplement: Supplementary file 2 — Supplementary file2 (DOCX 15 KB) [file 40279_2025_2331_MOESM2_ESM.docx]

Table 1. Anecdotal data from discussions with individuals that have experienced spinal cord stimulation

| Athlete One competed at the Tokyo 2020 Paralympics and shared their thoughts on electroceuticals and how they would have been useful when competing in the Games. They stated that their low resting BP led to an early onset of fatigue and impaired endurance, and the hot ambient temperatures combined with a lack of thermoregulatory heat loss mechanisms also predisposed them to exhaustion. They believed that an electroceutical such as SCS could have ameliorated these physiological impairments and they would have jumped at the chance to use one if they could.  Athlete Two participated in a research study using cardiovascular-optimised transcutaneous SCS to enhance upper-body exercise performance, in which they managed to exercise at a high-intensity for approximately 18 minutes longer than with the sham stimulation. They stated “As an already fit individual in the SCI community, this improvement in performance was phenomenal. The way my body felt between the two exercise trials, with and without stimulation, was truly remarkable. It was like I was given performance enhancing drugs while being electrically stimulated, not that I know what it is like to have ever taken performance enhancing drugs. But, with stimulation, I felt as though I had twice as much energy and could train twice as hard! I would be extremely intrigued to discover if a block of training with stimulation would improve my cardiorespiratory fitness and lead to greater performance times in my wheelchair racing events!” |
| --- |
